# Mechanism of enhanced salt tolerance in *Saccharomyces cerevisiae* by *CRZ1* overexpression

**DOI:** 10.1038/s41598-024-74174-1

**Published:** 2024-10-02

**Authors:** Furong Zuo, Yajing Wu, Yanqiu Sun, Caiyun Xie, Yueqin Tang

**Affiliations:** 1 College of Architecture and Environment, Sichuan University, Chengdu, 610065 Sichuan China; 2 Sichuan Environmental Protection Key Laboratory of Organic Wastes Valorization, Chengdu, 610065 Sichuan China; 3 Engineering Research Center of Alternative Energy Materials and Devices, Ministry of Education, Chengdu, 610065 Sichuan China

**Keywords:** Fuel ethanol, *Saccharomyces cerevisiae*, High-salt tolerance, Regulatory mechanism, Comparative transcriptomic analysis, Fungal host response, Applied microbiology, Metabolic engineering, RNA sequencing

## Abstract

Achieving high-gravity fermentation in the industrial production of fuel ethanol, and enhancing the fermentation efficiency of high-salt raw materials, such as waste molasses, can significantly reduce wastewater output and process costs. Therefore, the development of hyperosmotic-tolerant industrial *Saccharomyces cerevisiae* strains, capable of resisting high-salt stress, offers both environmental and economic benefits. Our previous study highlighted the potential of *CRZ1* overexpression as a strategy to improve the yeast strain’s resistance to high-salt stress, however, the underlying molecular mechanisms remain unexplored. The fermentation capabilities of the *CRZ1*-overexpressing strain, KCR3, and its parental strain, KF7, were evaluated under condition of 1.25 M NaCl at 35 °C. Compared to KF7, KCR3 showed an 81% increase in glucose consumption (129.25 ± 0.83 g/L) and a 105% increase in ethanol production (47.59 ± 0.93 g/L), with a yield of 0.37 g/g. Comparative transcriptomic analysis showed that under high-salt stress, KCR3 exhibited significantly upregulated expression of genes associated with ion transport, stress response, gluconeogenesis, and the utilization of alternative carbon sources, while genes related to glycolysis and the biosynthesis of ribosomes, amino acids, and fatty acids were notably downregulated compared to KF7. Crz1 likely expands its influence by regulating the expression of numerous transcription factors, thereby impacting genes involved in multiple aspects of cellular function. The study revealed the regulatory mechanism of Crz1 under high-salt stress, thereby providing guidance for the construction of salt-tolerant strains.

## Background

Bioenergy possesses characteristics of sustainability, low carbon emissions, and environmental friendliness^1^. In recent years, with the continuous depletion of fossil fuels and the growing prominence of environmental issues, the development of renewable and clean biofuels has become a vital mission in energy development. Relative to conventional gasoline, bioethanol features a higher octane number and is recognized as a highly promising bio-based liquid fuel. Microorganisms ferment glucose, xylose, and other sugars derived from cellulosic, saccharide, and starch feedstocks to produce ethanol. Achieving high-gravity fermentation and enhancing the fermentation efficiency of high-salt feedstocks, such as waste molasses, can significantly reduce wastewater output and lower processing costs. Moreover, developing production processes that use seawater as the medium can save freshwater resources. Therefore, the development of hyperosmotic ethanol-producing strains that can tolerate high-salt stress is of significant economic and environmental importance to the fuel ethanol industry.

Compared to other microorganisms, *Saccharomyces cerevisiae* (*S. cerevisiae*) stands out due to its benefits of biosafety, high ethanol productivity, rapid cell growth, robust environmental adaptability, and low nutrient requirements, making it a traditional organism for ethanol production. Some studies have acquired salt-tolerant strains using methods such as isolation from natural environments, mutagenesis and domestication^2–4^. Nonetheless, these strains do not produce ethanol, and breeding salt-tolerant strains remains a challenge because the mechanisms underlying their salt tolerance are not yet fully understood. Other studies have employed a combined strategy of omics and metabolic engineering to identify key targets that affect the salt tolerance of yeast^5–7^. For example, Matsushika et al.^8^ identified the *GAS1* gene from the genome of the multiple-stress-tolerant yeast, *Issatchenkia orientalis*. Upon overexpressing Io*GAS1* in *S. cerevisiae*, strain B4-IoGAS1 produced 17.1 g/L ethanol in the presence of 0.5 M Na_2_SO_4_, which is approximately 48.7% higher than its parental strain^9^. To date, the known salt-tolerant strains are still unable to cope with the high-salt stress encountered in the industrial process of fuel ethanol production. Therefore, further investigation into the mechanisms behind *S. cerevisiae*’s tolerance to high-salt stress, along with the identification of key genes influencing salt tolerance, is essential for the development of more robust salt-tolerant strains.

In our previous study, we successfully obtained a genetically stable, multi-tolerant strain designated E-158 through a combination of ARTP mutagenesis, genome rearrangement, and hybridization^10^. Notably, under 1.25 M NaCl, E-158 produced 56.01 ± 2.53 g/L ethanol, showing an improvement of 36% compared to its parental strain. The ethanol titer of E-158 under 1.25 M NaCl exceeded that of the B4-IoGAS1 strain under 0.5 M Na_2_SO_4_^9^. Furthermore, through comparative transcriptomics, genes beneficial for salt tolerance, such as *CRZ1*, were identified^11^. The transcription factor Crz1, encoded by *CRZ1*, serves as a primary target of calcineurin and is involved in regulating a set of genes that enable cells to adapt to stress conditions^12^. Yoshimoto et al.^13^ revealed that the calcineurin-Crz1 signaling pathway responds to Ca^2+^ or Na^+^ by regulating processes including ion transport, cell wall synthesis/maintenance, lipid and sterol metabolism, as well as vesicular transport. Stathopoulos et al.^14^ found that the overexpression of *CRZ1* increased the tolerance of yeast cells to Mn^2+^ and Li^+^.

Overall, the specific mechanisms by which *CRZ1* participates in the salt tolerance of *S. cerevisiae* are not yet fully understood. Most of the reported salt-tolerant strains remain at the laboratory stage and have not been applied to industrial-scale fuel ethanol production. In this study, we evaluated the salt tolerance of the *CRZ1*-overexpressing strain KCR3 under a 1.25 M NaCl condition. Through comparative transcriptomic analysis, we explored the molecular mechanisms by which Crz1 enhances high-salt tolerance. These findings serve as a guide for the development of salt-tolerant *S. cerevisiae* strains.

## Methods

### Strains and media

The industrial flocculating *S. cerevisiae* strain KF7^15^ and its derivative *CRZ1*-overexpressing strain KCR3^11^ were used in this study. Yeast cells were cultured on 2% YPD plates (YP medium (10 g/L yeast extract, 20 g/L peptone) with 20 g/L glucose and 15 g/L agar) for activation. For batch fermentation, yeast cells were pre-cultivated in 5% YPD medium (YP medium with 50 g/L glucose), and then fermented in 15% YPDN medium (YP medium with 150 g/L glucose and 1.25 M NaCl).

### Batch fermentation

Yeast cells were pre-cultivated aerobically at 30 °C and 200 rpm in 5% YPD medium for 14 h. Then, the cells were harvested and inoculated into 300 mL shake flasks, each containing 100 mL of 15% YPDN medium. The initial inoculum size was 0.47 g/L based on dry cell weight (DCW). Fermentation was performed at 35 °C in a thermostatic water bath with an agitation speed of 200 rpm. Samples were periodically taken to measure the DCW as well as the concentrations of glucose, ethanol, and glycerol. All experiments were performed in triplicate.

### Analytical methods

The fermentation broth underwent centrifugation at 8000 × g for 2 min. The precipitated cells were used for determining the dry cell weight. The supernatant was filtered through a 0.22 μm membrane filter before measuring the concentrations of glucose, ethanol, and glycerol. These analyses were conducted as previously described^16^. The concentrations of glucose and glycerol were assayed using HPLC equipped with a RID-10A refractive index detector (Shimadzu, Kyoto, Japan) and an Aminex HPX-87H column (Bio-Rad, Hercules, CA, USA). The concentration of ethanol was determined using gas chromatography (GC 353B, GL Sciences, Tokyo, Japan) with an FID detector. Isopropanol served as the internal standard. The yields of ethanol and glycerol were calculated based on the amount of glucose consumed.

### RNA extraction

Cells used for RNA extraction were collected at 48 h of fermentation under 1.25 M NaCl. Total RNA from three biological replicates of each strain was extracted using the Yeast RNA Kit (Omega Bio-Tek, Norcross, USA) following the manufacturer’s instructions. The quality and concentration of the total RNA were measured by Nanodrop 2000/2000C (Thermo Scientific, Waltham, USA) and confirmed through agarose gel electrophoresis. RNA-seq analysis was performed using Illumina NovaSeq X Plus by Novogene Technology Co. Ltd. (Beijing, China) according to the manufacturer’s recommendations. Approximately 6 Gb of clean data for each sample were used to perform the transcriptional analysis. The raw sequence data can be accessed through the SRA accession number PRJNA1114907.

### Transcriptome analysis

The comparative transcriptome analysis was conducted as previously described^17^. The gene annotation information was sourced from the Saccharomyces Genome Database (SGD, http://www.yeastgenome.org). Statistically significant differentially expressed genes (DEGs) were identified using the DESeq2 package in R software. The criteria for a DEG were an adjusted *p*-value (FDR) < 0.05 and a |log_2_(Fold change)|≥ 1. The DEGs were subjected to Gene Ontology (GO) and Kyoto Encyclopedia of Genes and Genomes (KEGG) enrichment analysis using the clusterProfiler R package. KEGG pathways were retrieved from the KEGG database (http://www.kegg.jp/kegg)^18^. The significance threshold was set to padj < 0.05. Protein–protein interaction networks (PPI) were analyzed using the STRING database (https://string-db.org/). Transcription factors (TFs) were identified through the YEASTRACT database (http://www.yeastract.com/index.php).

## Results and discussion

### Fermentation performance under high-salt conditions

**Fig. 1:**
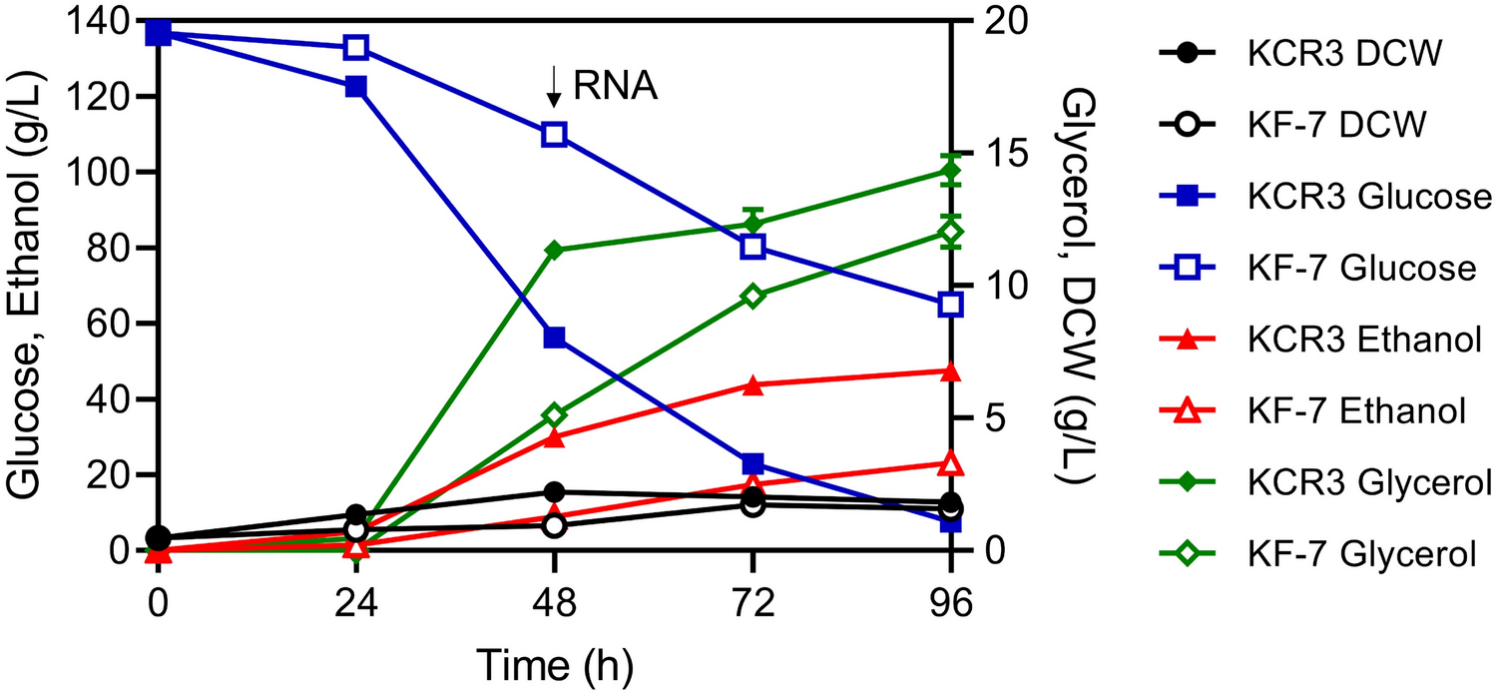
Fermentation profiles of strains KCR3 and KF7 in YP medium containing 150 g/L glucose and 1.25 M NaCl. The initial inoculum size was 0.47 g dry cell weight (DCW)/L. Symbols: ethanol (triangles), glucose (squares), DCW (circles), and glycerol (diamonds). Data are the means of triplicate experiments (error bars indicate standard deviation, SD).

The fermentation performance of strains KCR3 and KF7 was evaluated using glucose as the sole carbon source (Fig. 1). The two strains displayed identical fermentation characteristics when not exposed to NaCl (Supplementary file 1: Fig. S1). However, under a high-salt stress condition of 1.25 M NaCl, KCR3 showed significantly superior growth and ethanol production capability compared to KF7. After 96 h of fermentation, KF7 utilized 71.77 ± 2.33 g/L glucose and produced 23.20 ± 0.69 g/L ethanol with a yield of 0.32 g/g. Meanwhile, KCR3 consumed 129.25 ± 0.83 g/L glucose and generated 47.59 ± 0.93 g/L ethanol with a yield of 0.37 g/g. In comparison, KCR3 showed enhancements of approximately 81%, 105%, and 16% in glucose consumption, ethanol production, and ethanol yield, respectively, compared to KF7. Additionally, glycerol accumulated as a byproduct, with KCR3 showing a glycerol yield of 0.11 g/g, which is 35% lower than that of KF7 (0.17 g/g). These results indicated that overexpression of *CRZ1* can effectively enhance the strain’s tolerance to high-salt stress, suggesting that *CRZ1* is a key gene influencing the salt tolerance of *S. cerevisiae*. Currently, no salt-tolerant yeast strains suitable for industrial fuel ethanol production have been identified. Notably, the Io*GAS1*-overexpressing strain B4-IoGAS1exhibited favorable salt tolerance, achieving ethanol production of 17.1 g/L under 0.5 M Na_2_SO_4_^9^. By comparison, strain KCR3 displayed even more impressive salt tolerance under 1.25 M NaCl stress, establishing it as one of the most promising candidates for large-scale industrial fuel ethanol production thus far.

### Elucidating the salt tolerance mechanisms of KCR3

Comparative transcriptomic analysis was performed between KCR3 and KF7 under high-salt stress to elucidate the potential mechanisms by which *CRZ1* affects the salt tolerance of *S. cerevisiae*. Each strain had three biological replicates with good reproducibility (Supplementary file 1: Fig. S2). Compared to KF7, KCR3 had a total of 2254 DEGs, with 1063 genes notably upregulated and 1191 genes downregulated (Supplementary file 1: Fig. S3).

### GO enrichment analysis

GO enrichment analysis was conducted separately on the upregulated, downregulated, and all DEGs. Terms with padj < 0.05 were considered significantly enriched.

For the up-regulated DEGs, 26 GO terms were significantly enriched, comprising 21 Biological Process (BP), 3 Molecular Function (MF), and 2 Cellular Component (CC) (Supplementary file 2: Table S1). Notably, several of these enriched terms are related to regulation, such as biological regulation, regulation of transcription, regulation of metabolic process, regulation of biosynthetic process, and zinc ion binding. Additionally, terms such as macromolecule modification, cellular protein modification process, and protein modification process are related to modification. Furthermore, terms like integral component of membrane and intrinsic component of membrane, both related to membrane components, were also enriched. Remarkably, these enriched GO terms encompass only 184 of the DEGs, with 37 of these being TFs (Supplementary file 2: Table S2).

For the downregulated DEGs, 121 GO terms were significantly enriched, consisting of 97 BP, 15 MF, and 9 CC (Supplementary file 2: Table S3). Specifically, terms such as peptide biosynthetic process, translation, amide biosynthetic process, ribosome, ribonucleoprotein complex, non-membrane-bound organelle, structural molecule activity, structural constituent of ribosome, and aminoacyl-tRNA ligase activity are all associated with protein synthesis and ribosomal function. The enriched GO terms encompass 419 DEGs, with 11 of these being TFs (Supplementary file 2: Table S2).

For all DEGs, 69 GO terms were significantly enriched, including 55 BP, 7 MF, and 7 CC (Supplementary file 2: Table S4). These terms were entirely subsumed within the set of enriched terms identified for the downregulated DEGs. The enriched GO terms encompass a total of 569 DEGs, including 48 TFs (Supplementary file 2: Table S2).

### CRZ1 and regulatory processes

**Fig. 2:**
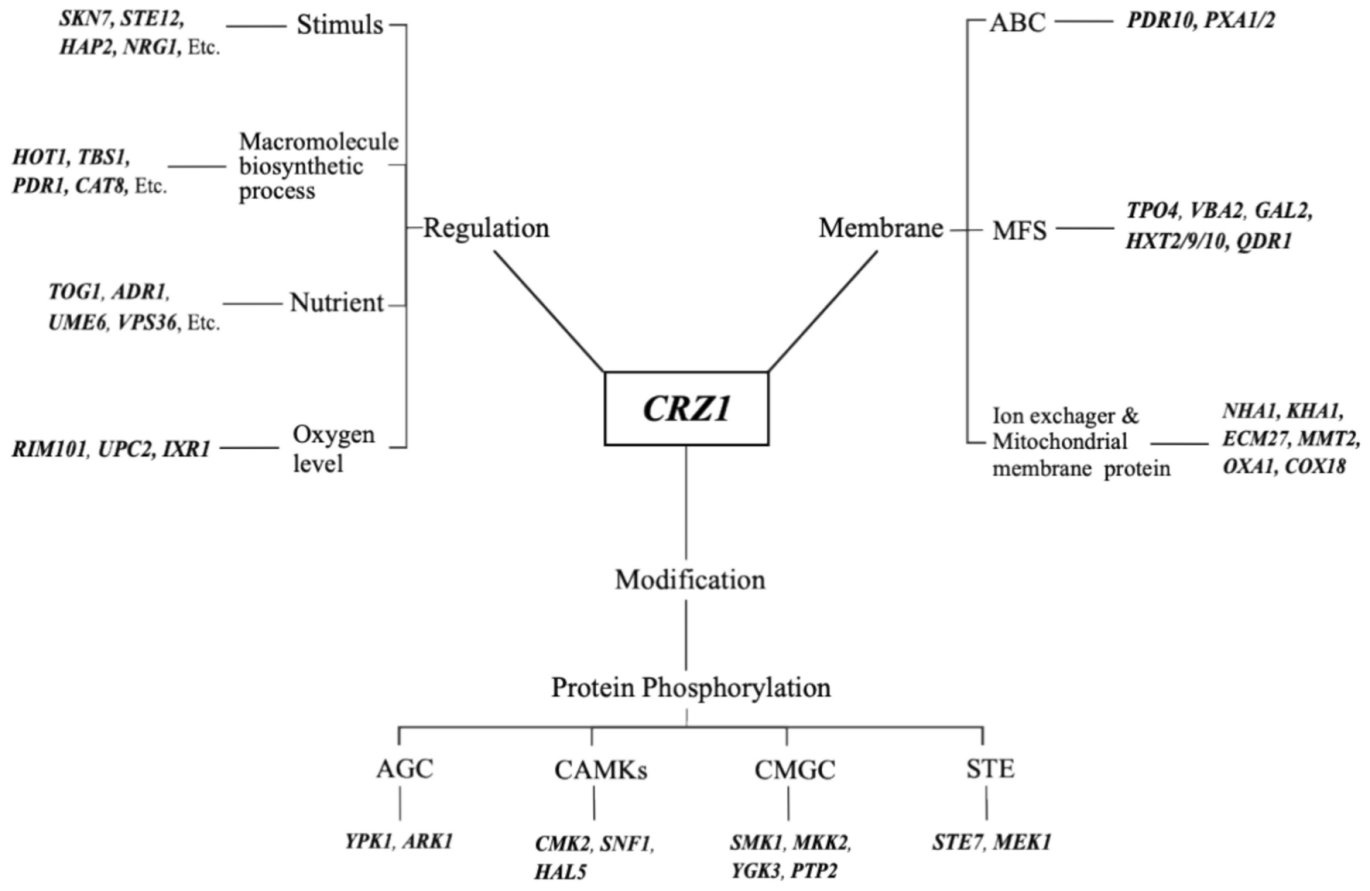
GO terms positively regulated by *CRZ1.*

Among the upregulated DEGs, 81 genes were involved in regulatory processes, including 37 TFs. From this, it is inferred that elevated *CRZ1* expression might amplify transcriptional signaling by modulating the expression of multiple TFs, offering a possible explanation for the large number of DEGs observed in KCR3 relative to KF7. Predominantly, these regulatory genes are engaged in regulating macromolecular biosynthesis (*HOT1*, *PDR1*, *CAT8*), responding to stress (*SKN7*, *STE12*, *NRG1*), adapting to nutrient limitation (*TOG1*, *ADR1*, *UME6)*, and coping with reduced oxygen levels (*RIM101*, *UPC2*, *IXR1*) (Fig. 2).

### CRZ1 and modification

Among the upregulated DEGs, a total of 54 genes were involved in protein modifications. Notably, 30 of these genes encode protein kinases (Supplementary file 2: Table S1). Here, we primarily focus on the impact of *CRZ1* on cellular protein phosphorylation processes. These protein kinases belong to various families, including AGC (Ypk1, Ark1), CAMKs (Cmk2, Snf1, Hal5), CMGC (Smk1, Mkk1, Ygk3, Ptp2), and STE (Ste7, Mek1) (Fig. 2). The AGC family is mainly related to cell membrane homeostasis, with Ypk1 serving as a central regulator of lipid and protein homeostasis at the plasma membrane^19^. Protein kinase Hal5 positively regulates the potassium transporters Trk1-Trk2 complex, thereby reducing membrane potential and minimizing Na^+^ uptake^20^. Smk1, Mkk1, Ygk3, Ptp2, and Mek1 are all implicated in meiosis and sporulation in diploid yeast^21–25^. Ste7 is involved in the pheromone-mediated mating response through G-protein coupled receptors^26^. Protein kinase Snf1 is essential for yeast to respond to glucose limitation and grow on non-fermentable carbon sources^27^, and its absence has been shown to increase cellular sensitivity to stresses such as Na^+^, alkaline pH, high temperature, and oxidative conditions^28^. The upregulation of these genes suggests that strain KCR3 may alter membrane fluidity and membrane potential to reduce Na^+^ uptake, adjust the cell cycle to induce sporulation, and enhance the utilization of non-fermentable carbon sources for energy supply to support these processes.

### CRZ1 and cell membrane

The ATP-binding cassette (ABC) superfamily (Pdr10, Pxa1/2), the major facilitator superfamily (MFS) (Tpo4, Vba2, Hxt2/9/10, Gal2, Qdr1), along with ion exchangers and mitochondrial membrane proteins (Ena1, Nha1, Kha1, Ecm27, Mmt2, Oxa1, Cox18) showed significant upregulation. ABC transporters utilize ATP hydrolysis to facilitate solute transport across membranes. Pdr10 plays a role in maintaining the normal distribution and function of membrane proteins^29^; Pxa1 and Pxa2, located in the peroxisomal outer membrane, are responsible for importing long-chain fatty acids (LCFAs) into peroxisomes for degradation^30^. MFS transporters take advantage of transmembrane electrochemical gradients to drive substance transport. Tpo4, a vacuolar membrane protein, participates in polyamine transport and recognizes spermidine and spermine^31^; The transporter Vba2 constitutes the major route for vacuolar transport of basic amino acids, enabling their recycling under nitrogen starvation conditions^32^; Hxt2 is a high-affinity glucose transporter that can also transport fructose and mannose, while Hxt 9/10 and Gal2 can transport glucose and galactose^33^. Furthermore, electrical membrane potential regulation is vital for intracellular cation homeostasis^20,34^. Under salt stress, cells need to expel Na^+^ efficiently. *ENA1* encodes a P-type ATPase capable of exporting toxic Na^+^ in the absence of a H^+^ gradient^35^; The K^+^ (Na^+^)/H^+^ antiporter encoded by *NHA1* mediates active sodium efflux^36^. The putative plasma membrane K^+^/H^+^ antiporter Kha1, situated in the Golgi apparatus, contributes to regulating cytosolic cation concentrations and maintaining pH stability^37^. *ECM27* encodes a Na^+^/Ca^2+^ exchanger located on the endoplasmic reticulum (ER) membrane, which plays a role in maintaining calcium homeostasis, regulating intracellular trehalose levels, and controlling cell cycle progression^38^. Saeki et al.^39^ found that Ca^2+^ supplementation under salt stress aids in maintaining normal mitochondrial function and suppresses reactive oxygen species (ROS) generation. Mmt2, a cation diffusion facilitator located in mitochondria, exports iron from the mitochondria to the cytoplasm, where it reacts with superoxide to protect the cell from oxidative damage^40^. Oxa1 is crucial for the correct assembly of mitochondrial respiratory chain complexes, and Cox18 is required for the proper assembly of cytochrome oxidase^41^.

**Fig. 3:**
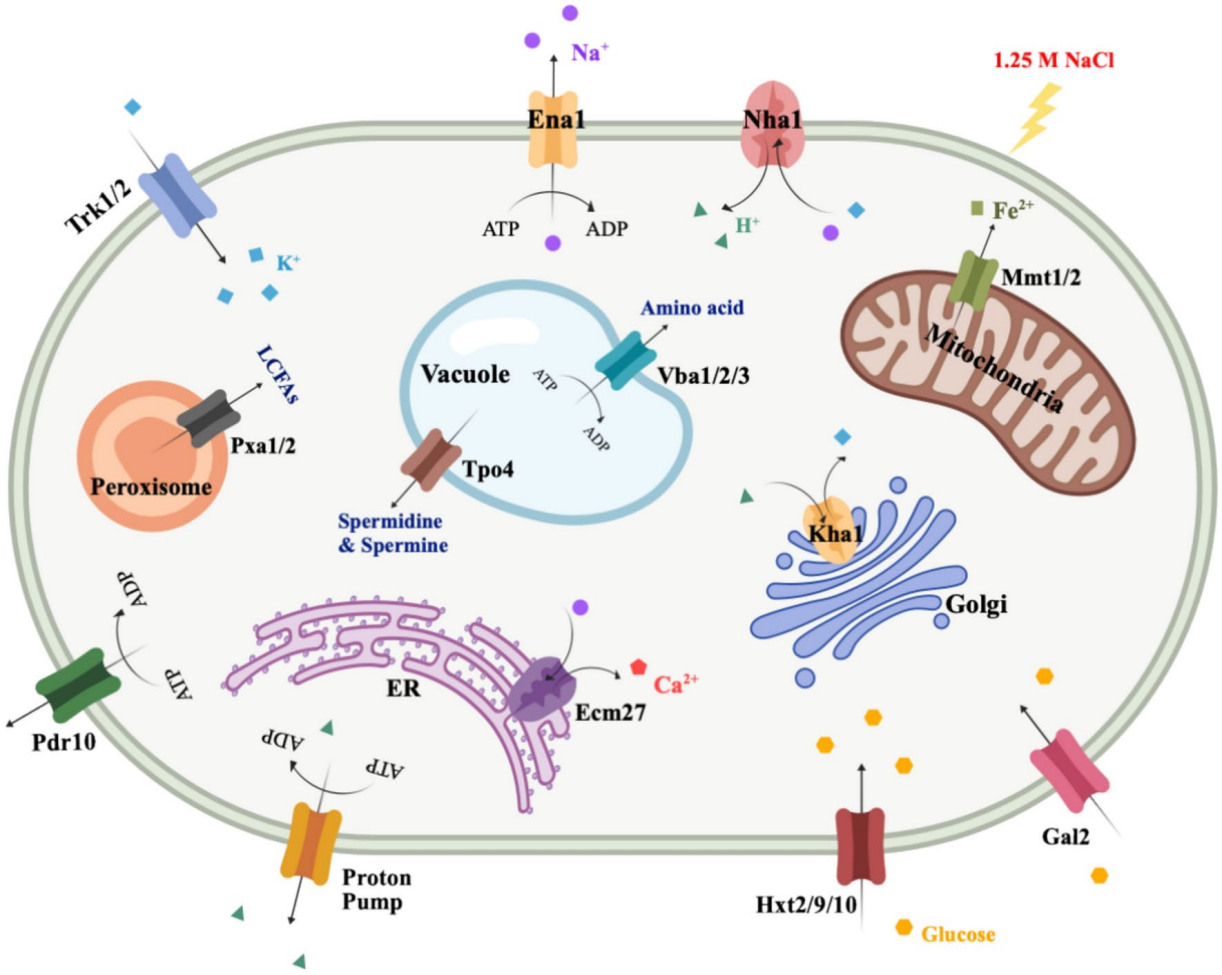
Various membrane proteins positively regulated by Crz1.

Compared to KF7, KCR3 exhibited a significant upregulation of genes related to protein assembly, substance transport, and ion exchange on both plasma and organellar membranes. We speculate that *S. cerevisiae* might employ the following strategies to cope with high-salt stress (Fig. 3): Firstly, the preservation of membrane integrity is prioritized, achieved through the proper assembly of membrane proteins and the prompt clearance of impaired plasma membrane components. Secondly, by enhancing the absorption of alternative cations such as K^+^ and Ca^2+^, the membrane potential is decreased, thereby reducing Na^+^ influx. Lastly, efficient expulsion of intracellular Na^+^ is facilitated by K^+^ (Na^+^)/H^+^ antiporters. Considering the energetic demands of these processes, the cell is likely to augment sugar uptake and the utilization of fatty acids to guarantee an adequate energy supply. Moreover, given the pivotal role of mitochondria in cellular respiration, ensuring the functional integrity of inner membrane enzymes and protecting them from oxidative damage is of great importance.

### KEGG enrichment analysis and PPI analysis

KEGG enrichment analysis was performed separately for the upregulated, downregulated, and all DEGs. Pathways with an adjusted padj < 0.05 were considered as significantly enriched pathways.

**Fig. 4:**
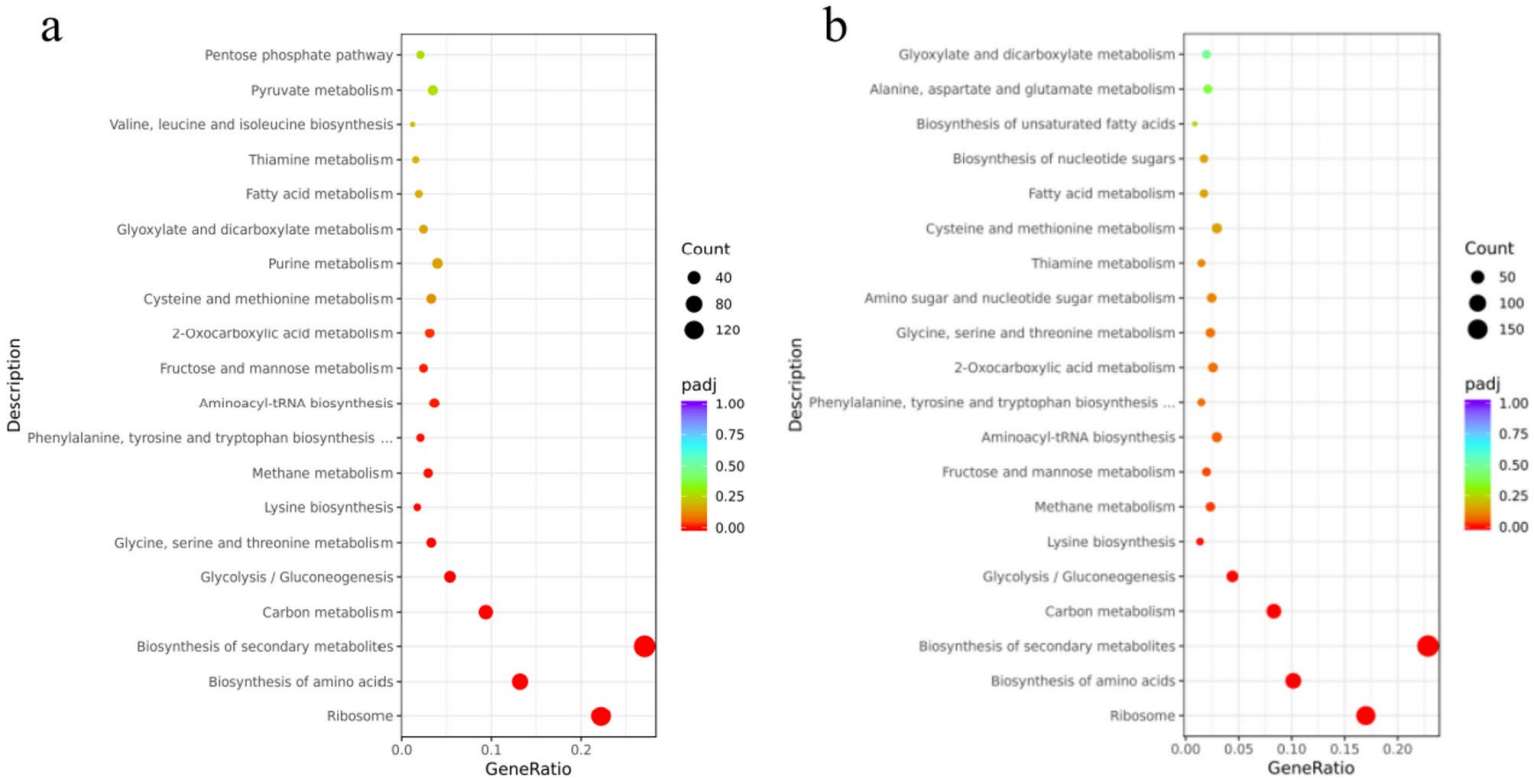
KEGG enrichment analysis of downregulated (**a**) and all (**b**) DEGs between KCR3 and KF7 under 1.25 M NaCl stress.

For the upregulated DEGs, no significantly enriched pathways were identified. For the downregulated DEGs, pathways including ribosome, biosynthesis of amino acids, carbon metabolism, glycolysis/gluconeogenesis, and aminoacyl-tRNA biosynthesis were significantly enriched (Fig. 4a, Supplementary file 2: Table S5). For all DEGs, pathways such as ribosome, biosynthesis of amino acids, biosynthesis of secondary metabolites, carbon metabolism, glycolysis, and lysine biosynthesis were notably enriched (Fig. 4b, Supplementary file 2: Table S6).

**Fig. 5:**
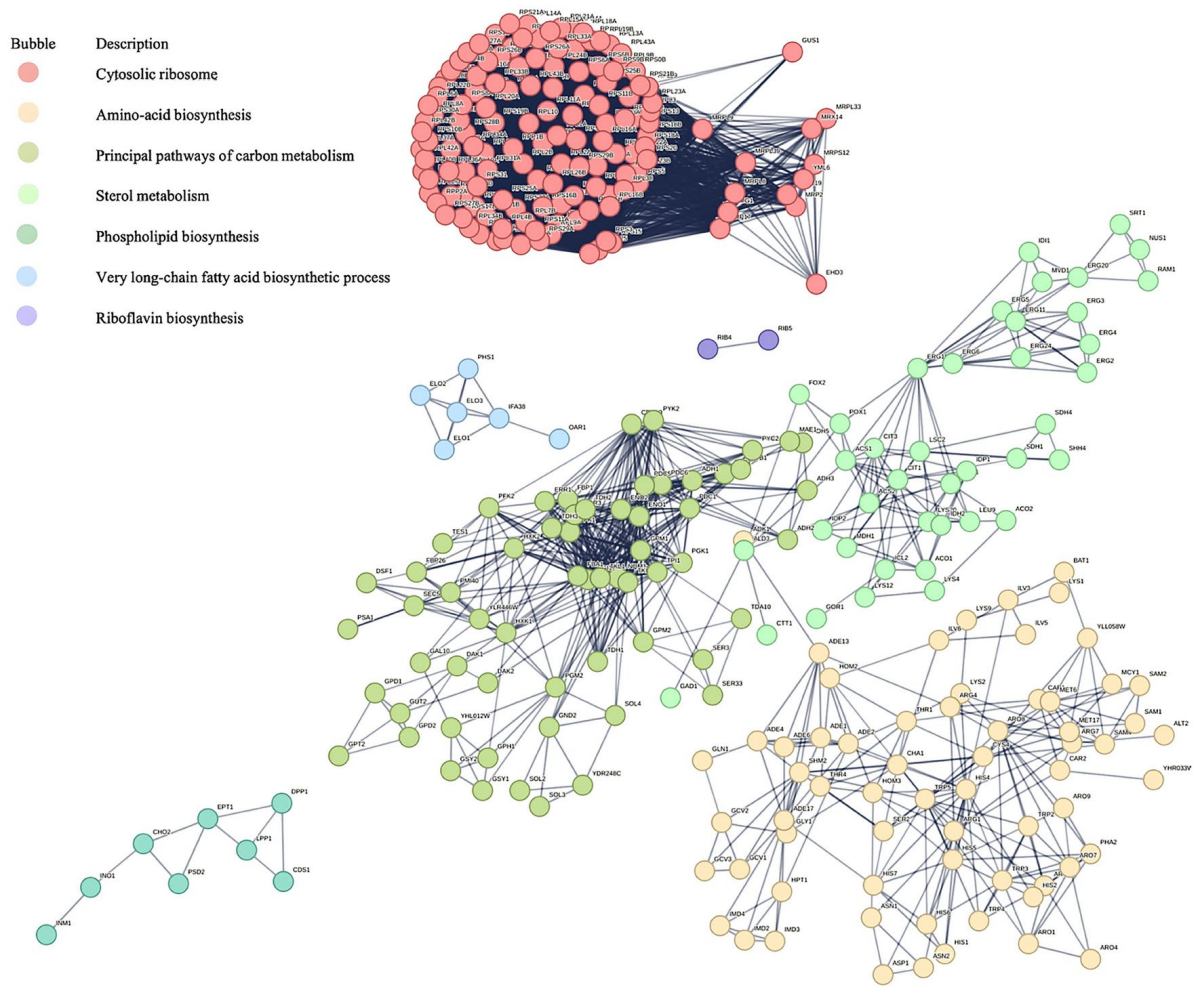
PPI analysis of DEGs in significantly enriched pathways.

**Fig. 6:**
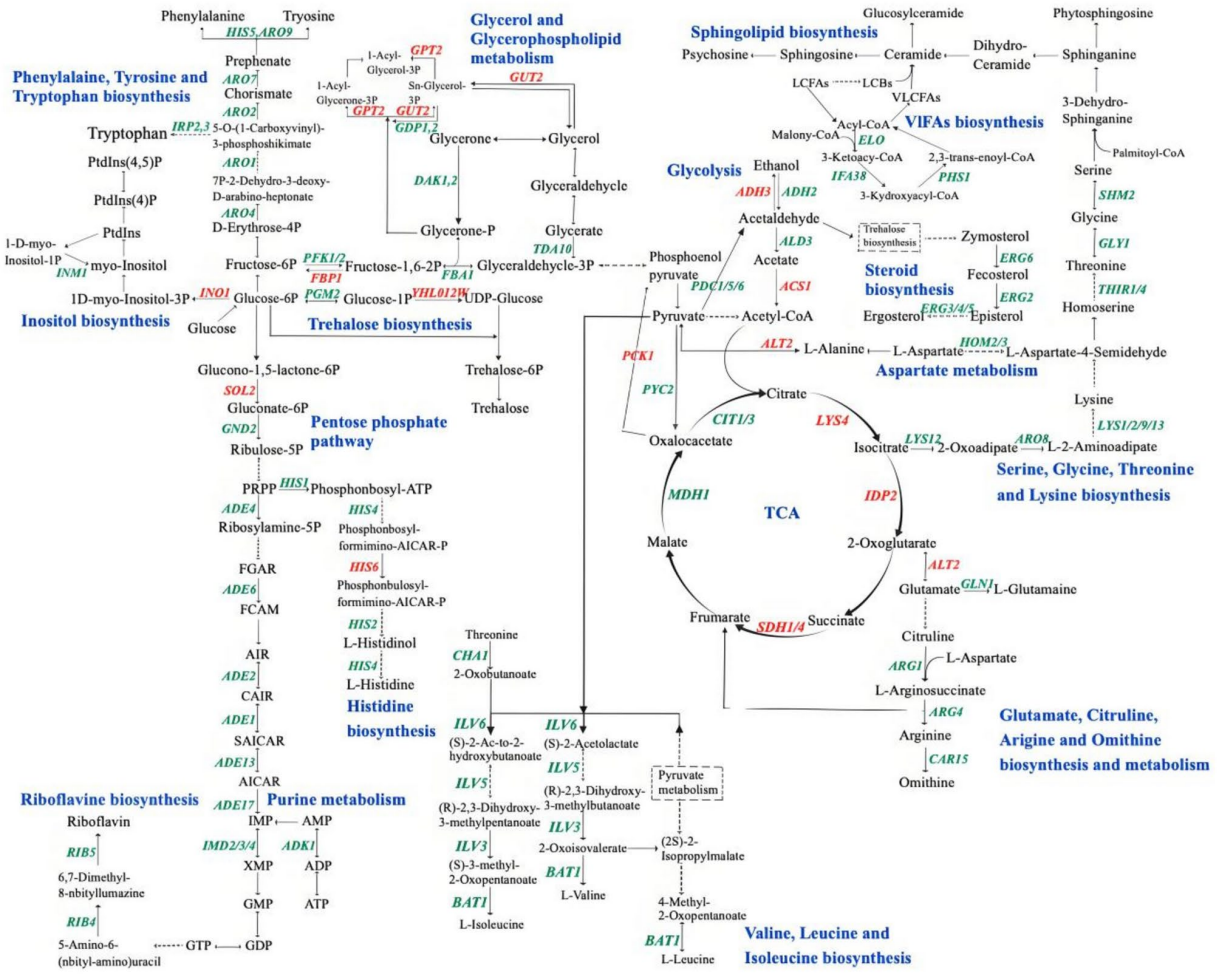
Significantly altered metabolic pathways in KCR3 compared to KF7 under high-salt stress. Red indicates upregulation, green indicates downregulation.

To investigate the relationships among genes in the enriched pathways, PPI analysis was conducted for the DEGs covered in the significantly enriched pathways. As shown in Fig. 5 and Supplementary file 2: Table S7, these genes were clustered into seven groups: cytosolic ribosome, amino acid biosynthesis, principal pathways of carbon metabolism, sterol metabolism, phospholipid biosynthesis, very long-chain fatty acid (VLCFA) biosynthetic process, and riboflavin biosynthesis. To further understand the high-salt tolerance mechanisms of *S. cerevisiae*, we conducted analyses and discussions focusing on key pathways and gene clusters (Fig. 6).

### Ribosome

Most genes associated with ribosomes are downregulated in KCR3, except for *EHD3*, *IMG1*, *YML6*, *MRX14*, *MRP2/17*, *MRPL8/9/19/33/39*, and *MRPS12*. *EHD3* encodes a 3-hydroxyisobutyryl-CoA hydrolase that catalyzes valine degradation, which ultimately feeds into the tricarboxylic acid (TCA) cycle for energy production. Meanwhile, *IMG1*, *YML6*, *MRX14*, *MRP2/17*, *MRPL8/9/19/33/39*, and *MRPS12* all code for mitochondrial ribosomal proteins integral to cellular respiration. This observation suggests that under high-salt stress, yeast cells may reduce cytosolic ribosome biosynthesis while enhancing mitochondrial respiration as a strategy to generate more energy to resist the adverse conditions.

### Amino acid biosynthesis

Most DEGs related to amino acid biosynthesis were significantly downregulated, including the biosynthesis of lysine (*LYS1/2/9*, *HOM2/3*, *ARO8*), arginine (*ARG1/4/7*, *CAR1*, *GLN1*), histidine (*HIS1/2/4/7*), valine, leucine, and isoleucine (*ILV3/5/6*, *BAT1*, *CHA1*), as well as phenylalanine, tyrosine and tryptophan (*ARO1/2/4/7/8/9*, *TRP2/3/4/5*, *HIS5*, *PHA2*). *S. cerevisiae* appears to mitigate amino acid biosynthesis to conserve energy in response to high-salt stress. Concurrently, the cell is likely to enhance the recycling and reuse of these amino acids. The observed upregulation of the amino acid transporter Vba2 on the vacuolar membrane supports this hypothesis.

### Carbon metabolism

In KCR3, most genes involved in glycolysis and the pentose phosphate pathway (PPP) were significantly downregulated, whereas genes involved in the TCA cycle (*LYS4*, *IDP2*, *SDH1/4*), glyoxylate cycle (*ICL2*), gluconeogenesis (*FBP1/26*, *PCK1*), and UDP-glucose synthesis (*GAL10*, *YHL012W*) were significantly upregulated. The upregulation of genes in the TCA cycle and glyoxylate cycle may enhance cellular respiration and energy production. The enzyme encoded by *GAL10* promotes the interconversion between UDP-galactose and UDP-glucose, while the enzyme encoded by *YHL012W* catalyzes the synthesis of UDP-glucose, serving as a precursor for trehalose synthesis. Additionally, genes related to glycerol synthesis (*GPD1/2* and *GPP1/2*) were significantly downregulated, whereas genes involved in glycerol catabolism (*GPT2* and *GUT2*) were significantly upregulated, potentially leading to a reduction in glycerol levels within KCR3. Fermentation results confirmed that under high-salt stress, KCR3 exhibited a lower glycerol yield compared to KF7, consistent with this observation. In the ethanol synthesis pathway, the key ethanol-producing gene *ADH3* was significantly upregulated, whereas the ethanol-consuming genes (*ADH2* and *ALD3*) were significantly downregulated. This was consistent with the observed phenotype of elevated ethanol titer and yield in KCR3.

### Biosynthesis of sterols and phospholipids

Ergosterol, the principal sterol constituent in *S. cerevisiae*, governs membrane fluidity, membrane protein activity, and transport capability. In KCR3, genes related to ergosterol biosynthesis (*ERG2/3/4/5/6*) were significantly downregulated, possibly resulting in altered membrane fluidity. Moreover, the inositol-3-phosphate synthase gene *INO1* was significantly upregulated, whereas the inositol monophosphatase gene *INM1* was significantly downregulated. This shift may facilitate the conversion of myo-inositol into phosphatidylinositol 4,5-bisphosphate (PIP2). As a key component of phospholipids, an increase in PIP2 content is crucial for the regulation of various signal transduction pathways and cellular functions^42^.

### Very long-chain fatty acids biosynthesis

Genes linked to VLCFA biosynthesis, including *ELO1/2/3*, *IFA38*, and *PHS1*, were significantly downregulated. *ELO1/2/3* encode fatty acid elongases, and *PHS1* codes for a 3-hydroxyacyl-CoA dehydrogenase, both central to fatty acid elongation. The microsomal β-ketoreductase encoded by *IFA38* participates in VLCFA synthesis. Reduced expression of these genes implies diminished VLCFA synthesis, possibly decreasing sphingolipid levels in cellular membranes. Sphingolipids, fundamental components of cellular membranes, play roles in cell growth and stress responses^43^. Their structural core, ceramides, act as stress sensors, participating in the regulation of cell cycle, endocytosis, and protein trafficking^44^. Kihara et al.^45^ reported that mutations in VLCFA synthesis genes led to a decline in complex sphingolipids and an increase in ceramides. It is thus speculated that KCR3 might augment intracellular ceramide levels by reducing VLCFA synthesis, thereby enhancing its capacity to withstand high-salt stress.

### Riboflavin biosynthesis

Genes involved in purine biosynthesis (*ADE1/2/4/6/13/17*, *IMD2/3/4*, *ADK1*, *HPT1*) were all downregulated in KCR3, which may lead to a decrease in intracellular GTP production. GTP serves as a precursor for riboflavin synthesis, a process catalyzed by enzymes encoded by *RIB4* and *RIB5*. The downregulation of *RIB4* and *RIB5* results in reduced riboflavin levels, which may further impact the contents of its derivative, flavin adenine dinucleotide (FAD), thereby affecting the function of the electron transport chain^46^. Lynch et al.^47^ observed that strains with lowered FAD levels exhibited increased alcohol dehydrogenase (ADH) activity. Our experimental data align with this trend, as evidenced by the upregulation of *ADH3* in KCR3.

### Interaction analysis of Crz1 with other significantly different expressed TFs

As mentioned above, Crz1 is postulated to indirectly control the expression of target genes by regulating their associated TFs. To elucidate the regulatory mechanisms by which Crz1 governs the salt tolerance of *S. cerevisiae*, we conducted an analysis focusing on TFs among all DEGs. In total, 2254 DEGs were regulated by 223 TFs (Supplementary file 2: Table S8), with 78 of these TFs showing significant differential expression under high-salt stress (Supplementary file 2: Table S9). Importantly, 2154 of the DEGs have been experimentally validated to be under the regulation of these 78 TFs, representing 95.56% of all DEGs (Supplementary file 2: Table S10). This finding reinforces our hypothesis that Crz1 expands its regulatory impact by modulating other TFs, ultimately leading to a marked enhancement in the salt tolerance of *S. cerevisiae*. Indeed, it is plausible that Crz1 and these other TFs exert yet unexplored regulatory effects on an even broader range of genes.

### TFs directly interacting with Crz1

**Fig. 7:**
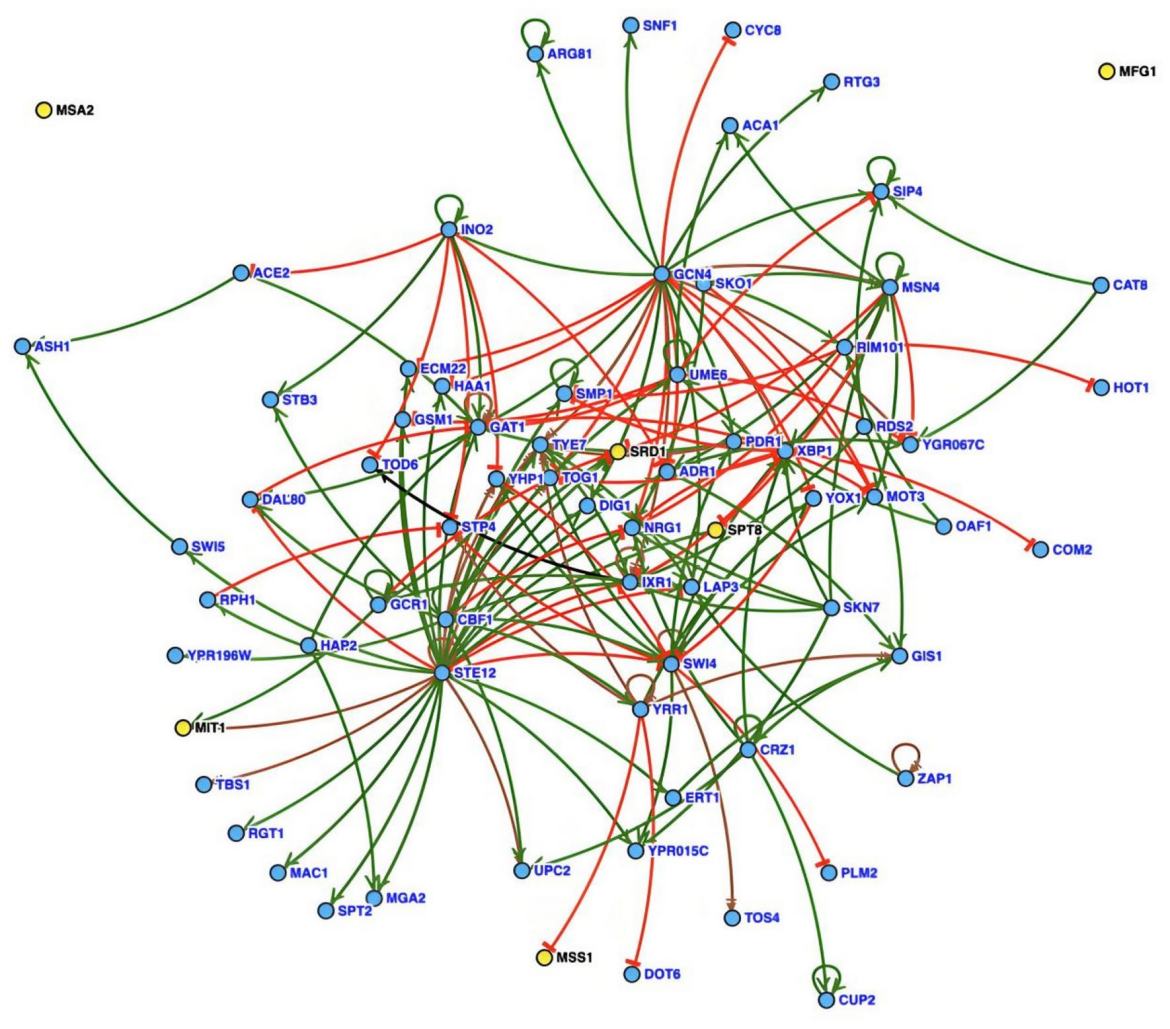
Regulatory networks of significantly differentially expressed TFs interacting with Crz1. Green indicates activation, red indicates inhibition, and brown indicates both activation and inhibition.

Analysis of the regulatory network involving 78 differentially expressed TFs indicated that Crz1 directly activates Cup2, Upc2, Xbp1, Nrg1, and Gis1 (Fig. 7, Supplementary file 2: Table S11). These five TFs play pivotal roles in regulating the cell’s resistance to adverse conditions. Specifically, Cup2 triggers the expression of the copper-zinc superoxide dismutase gene *SOD1* to eliminate intracellular ROS^48^. Upc2 activates genes responsible for cell wall mannoproteins, thus preserving the cell wall’s osmotic stability^49^. Xbp1 suppresses transcription of G1-specific cyclins, promoting sporulation as a strategy against harsh environments^50^, and also initiates the unfolded protein response (UPR) to maintain endoplasmic reticulum (ER) homeostasis^51^. Nrg1 reacts to stresses such as high-salt, alkaline pH and nutrient limitation^52^, contributing to the regulation of filamentation, invasive growth, and sporulation of the strain^53^. Gis1 facilitates the cell’s timely recognition of environmental shifts^54^, and further induces spore wall biosynthesis^55^. Significantly, all five TFs, along with Crz1, were significantly upregulated. Correspondingly, the marked upregulation of sporulation genes *SMK1*, *MKK1*, *YGK3*, and *PTP2* suggests that the cell may enhance its salt tolerance by facilitating sporulation.

### TFs indirectly interacting with Crz1

A considerable amount of glucose was still present in the medium when cells were collected at 48 h for RNA isolation (Fig. 1). However, some TFs related to the utilization of non-fermentable carbon sources were significantly upregulated. Snf1 is essential for yeast cell growth on non-fermentable carbon sources^27^. Adr1 and Cat8 are under the regulation of Snf1^56^. Oaf1 facilitates the utilization of fatty acids^57^, and Tog1 induces the expression of genes related to fatty acid β-oxidation, NADPH regeneration, and gluconeogenesis^58^. In this study, these TFs, along with genes related to fatty acid β-oxidation (*POX1*, *FOX2*, *ECI1*, *PXA1/2*), were notably upregulated in KCR3 when compared to KF7. These results suggested that KCR3 may harness the fatty acid β-oxidation process for energy production under high-salt stress.

The alleviation of nitrogen catabolite repression (NCR) ensures that cells can utilize non-preferred or alternative nitrogen sources^59^. The transcriptional activator Gat1 and the repressor Dal80 jointly control the NCR pathway^60^. In this study, the notable upregulation of *GAT1* and the downregulation of *DAL80* implied that the strain may have promoted the expression of NCR-sensitive genes. Consequently, the allantoin degradation gene *DAL1*, amino acid catabolism genes *CHA4* and *ASP3-3*, and genes encoding ammonium and amino acids transmembrane transporters (*MEP1*, *ALP1* and *BAP2*) all showed increased expression. These observations indicated that relief from NCR may facilitate the adaptation to high-salt stress.

### Summary of the *CRZ1* regulatory mechanisms

**Fig. 8:**
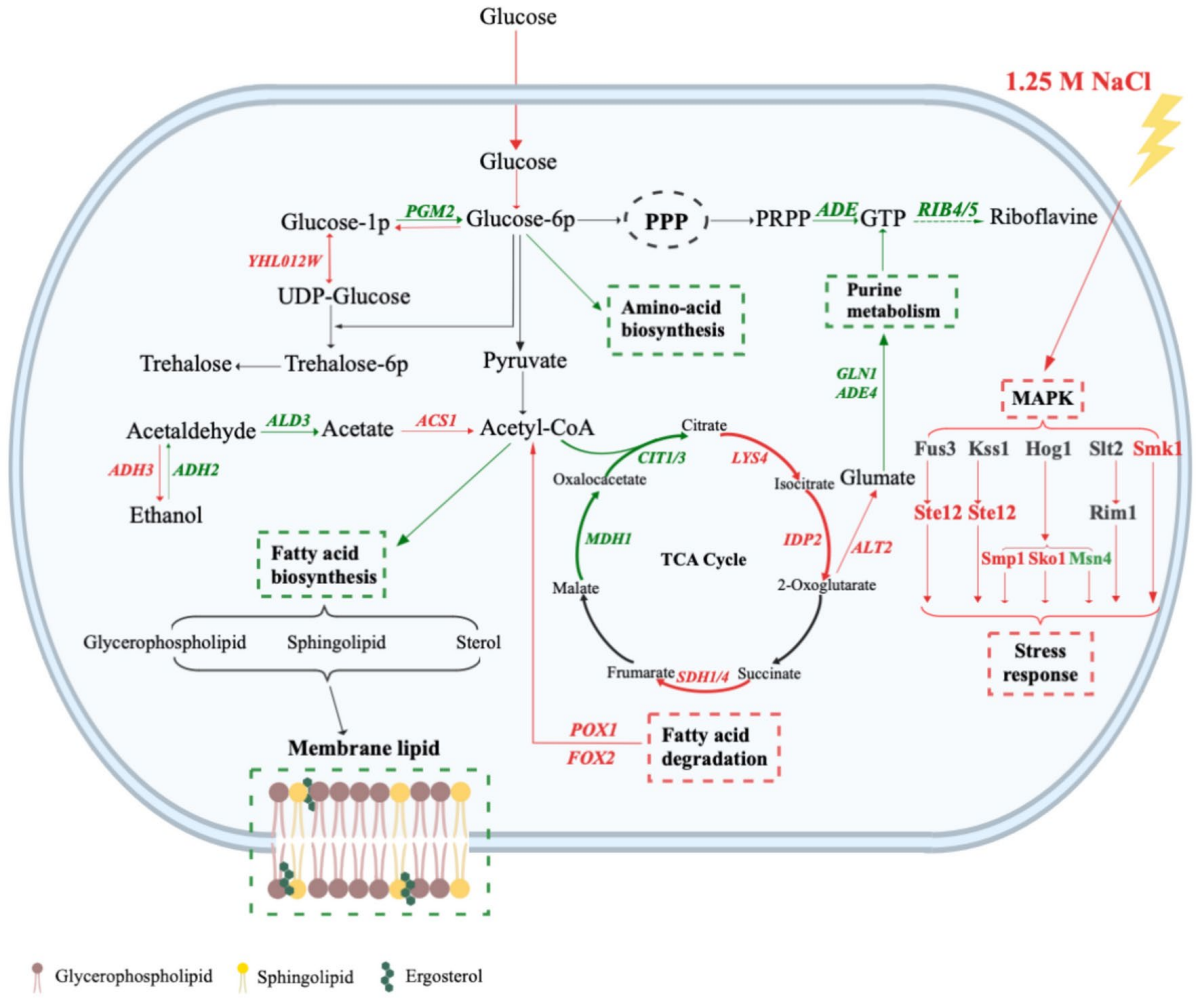
Illustration of the salt tolerance mechanism of *CRZ1*-overexpressing strain KCR3 under 1.25 M NaCl. Red indicates upregulation and green indicates downregulation.

Crz1 potentially extends its regulatory impact to a broader range of genes by regulating multiple TFs, thereby indirectly controlling their downstream target genes. In this study, we speculated that Crz1 copes with high-salt stress by activating pathways related to ion transport, stress responses, and fatty acid utilization, while concurrently inhibiting glycolysis and suppressing the biosynthesis of ribosomes, amino acids and fatty acids (Fig. 8).

Ion transport is crucial for maintaining intracellular ionic homeostasis. On one hand, cells enhance the uptake of cations such as K^+^ and Ca^2+^ to decreases membrane potential, which in turn reduces Na^+^ influx. On the other hand, cells utilize Na^+^/K^+^-ATPases and K^+^ (Na^+^)/H^+^ antiporters to expel excess Na^+^ from the cytoplasm, effectively lowering intracellular Na^+^ levels. Furthermore, a marked upregulation of genes and TFs related to sporulation in KCR3 suggested that this strain adjusts its cell cycle to enhance tolerance to high-salt stress. These findings align with previous research, which revealed that cells respond to salt and hyperosmotic stress by modulating transporter functions at the plasma membrane and adjusting the progression of the cell cycle^61,62^.

In contrast to the genes implicated in cytoplasmic ribosome biosynthesis, those involved in ribosome biosynthesis inside mitochondria exhibited elevated expression. Moreover, several genes related to fatty acids β-oxidation were notably upregulated in KCR3. Studies have revealed that upon salt stress, the activation of mitochondrial respiration relies on peroxisomes, which supply acetyl-CoA to the mitochondria^63,64^. Consequently, Crz1 potentially contributes to cellular salt tolerance by stimulating the synthesis of mitochondrial ribosomes and the provision of acetyl-CoA through fatty acid oxidation.

## Conclusion

The study entailed a comparative transcriptomic analysis between the *CRZ1*-overexpressing strain, KCR3, and its parental strain, KF7, under high-salt stress. The results unveiled that Crz1 employed a multifaceted regulatory approach to enable cells to overcome high-salt stress. More specifically, Crz1 interacts with a multitude of transcription factors, thereby exerting indirect influence on the transcription of numerous downstream genes. Its potential mechanisms for conferring salt tolerance include modulating the function of cell membrane proteins, reducing cytoplasmic translation efficiency, repressing the synthesis of macromolecules, initiating the use of non-fermentative carbon sources and alternative nitrogen sources, activating mitochondrial respiration, and facilitating sporulation. The findings of this study contribute to a deeper understanding of the mechanisms by which *CRZ1* regulates salt tolerance, thereby providing direction for the development of robust, industrially applicable, salt-tolerant yeast strains.

## Supplementary Information

Supplementary Information 1.

Supplementary Information 2.

## Data Availability

The transcriptome datasets analyzed in this study can be accessed through the SRA accession number PRJNA1114907.

## Abbreviations

*S. cerevisiae*: *Saccharomyces cerevisiae*
DEGs: Differentially expressed genes
GO: Gene ontology
BP: Biological process
MF: Molecular function
CC: Cellular component
KEGG: Kyoto encyclopedia of genes and genomes
SGD: Saccharomyces genome database
TFs: Transcription factors
RNA: Ribonucleic acid
ABC: ATP-binding cassette
LCFAs: Long-chain fatty acids
MFS: Major facilitator superfamily
ROS: Reactive oxygen species
PPI: Protein–protein interaction networks
PIP2: Phosphatidylinositol 4,5-bisphosphate
VLCFA: Very long-chain fatty acid
ATP: Adenosine-triphosphate
TCA cycle: Tricarboxylic acid cycle
PPP: Pentose phosphate pathway
FAD: Flavin adenine dinucleotide
ADH: Alcohol dehydrogenase
UPR: Unfolded protein response
ER: Endoplasmic reticulum
NCR: Nitrogen catabolite repression
DCW: Dry cell weight
GC: Gas chromatography
HPLC: High-performance liquid chromatography
FID: Flame ionization detector
NADPH: Nicotinamide adenine dinucleotide phosphate
