# Supplementary material for: Mechanism of enhanced salt tolerance in Saccharomyces cerevisiae by CRZ1 overexpression
Source: Sci Rep. 2024 Oct 2;14:22875. doi: 10.1038/s41598-024-74174-1 (PMC11447063; doi:10.1038/s41598-024-74174-1)
Supplement: Supplementary file 1 — Supplementary Information 1. [file 41598_2024_74174_MOESM1_ESM.docx]

**Mechanism of enhanced salt tolerance in *Saccharomyces cerevisiae* by *CRZ1* overexpression**

Furong Zuo^1, 2^, Yajing Wu^1, 2^, Yanqiu Sun^1, 2^, Caiyun Xie^1, 2, 3^*, Yueqin Tang^1, 2, 3^

**Supplementary file 1 - content**

**Fig. S1** Fermentation profiles of strains KCR3 and KF7 in YP medium containing 150 g/L glucose. The initial inoculum size was 0.47 g dry cell weight (DCW) /L. Symbols: ethanol (triangles), glucose (squares), and glycerol (diamonds). Data are the means of triplicate experiments (error bars indicate standard deviation, SD).

**Fig. S2** Heat map of gene clustering

**Fig. S3** Volcano plot of DEGs between KCR3 and KF7 under 1.25 M NaCl

**Fig. S1** Fermentation profiles of strains KCR3 and KF7 in YP medium containing 150 g/L glucose. The initial inoculum size was 0.47 g dry cell weight (DCW)/L. Symbols: ethanol (triangles), glucose (squares), and glycerol (diamonds). Data are the means of triplicate experiments (error bars indicate standard deviation, SD).


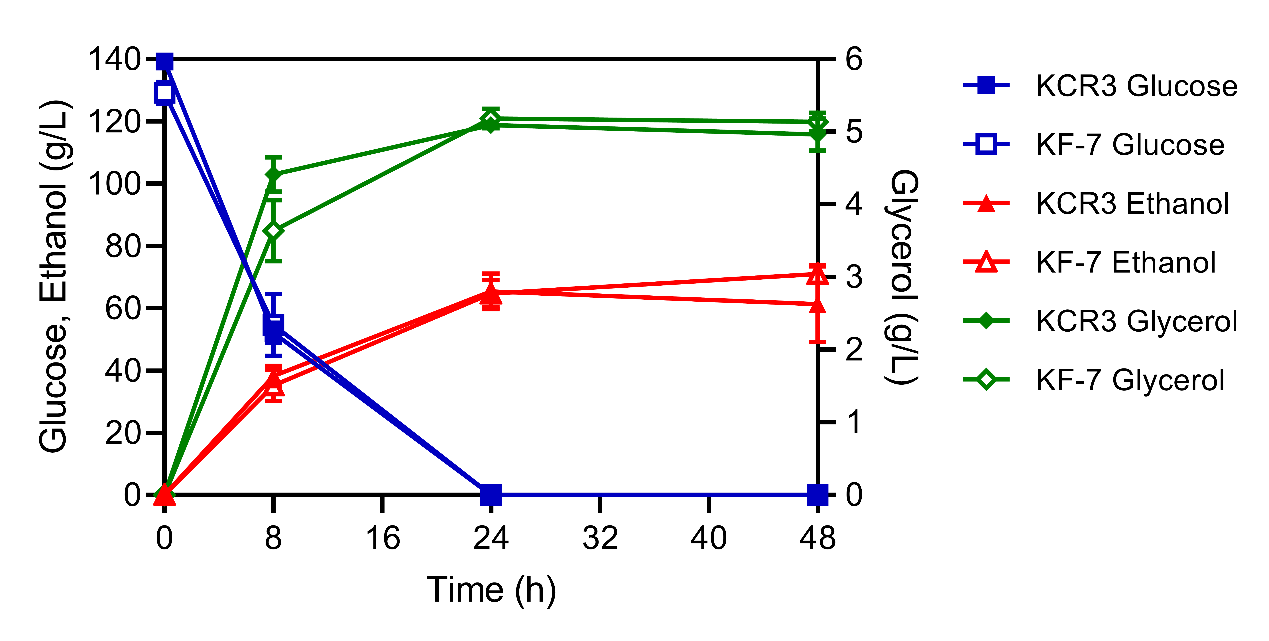


**Fig. S2** Heat map of gene clustering


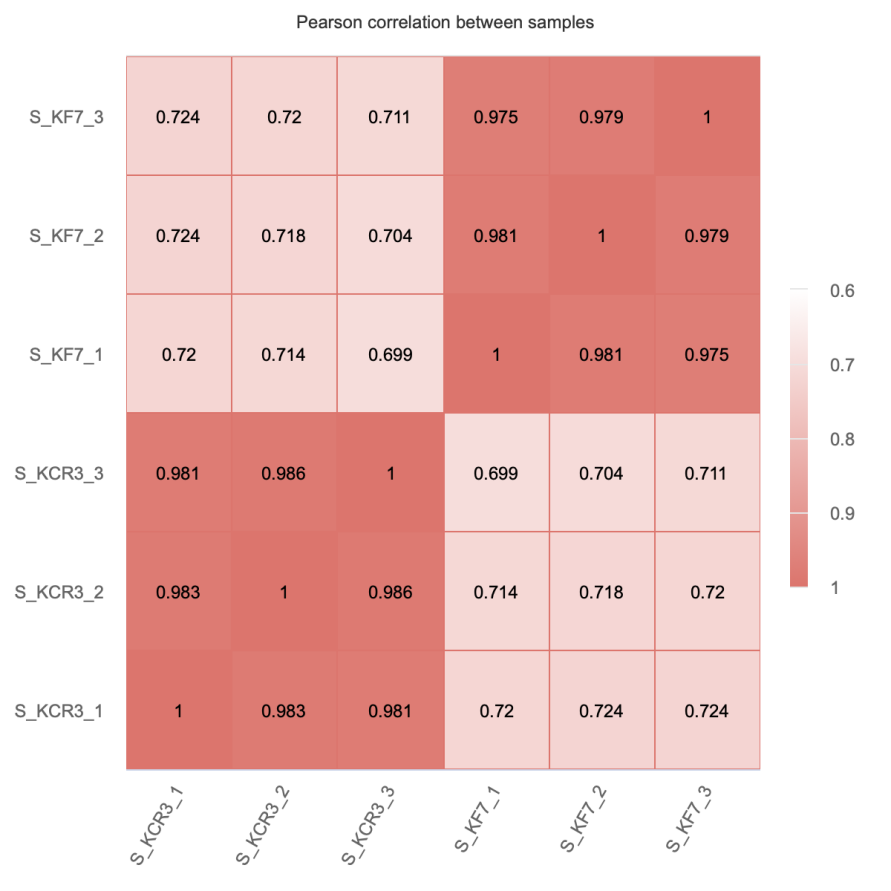


**Fig. S3** Volcano plot of DEGs between KCR3 and KF7 under 1.25 M NaCl

**
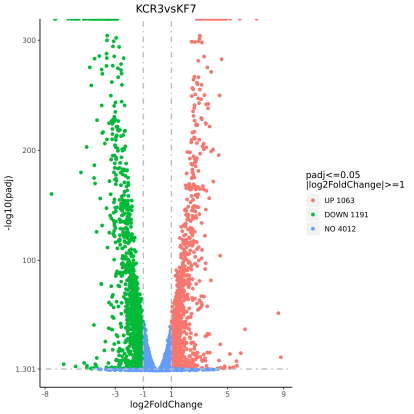
**
